# Out-of-clinic and self-managed abortion in Bangladesh: menstrual regulation provider perspectives

**DOI:** 10.1186/s12978-021-01123-w

**Published:** 2021-03-25

**Authors:** Bonnie Crouthamel, Erin Pearson, Sarah Tilford, Samantha Hurst, Dipika Paul, Fahima Aqtar, Jay Silverman, Sarah Averbach

**Affiliations:** 1 Department of Obstetrics, Gynecology, and Reproductive Sciences, University of California San Diego, 9300 Campus Point Drive, La Jolla, San Diego, CA 92037 USA; 2 Ipas, P.O. Box 9990, Chapel Hill, NC 27515-7052 USA; 3 Department of Family Medicine and Public Health, University of California San Diego, 9500 Gilman Drive #0507 La Jolla, San Diego, CA 92093-0507 USA; 4 Ipas Bangladesh, Eureka Saleha Palace, (Flat-B2 and C2), 2nd Floor, House #2F-1-3, Mymensingh Road, Shahbag, Dhaka 1000 Bangladesh; 5 Center on Gender Equity on Health, School of Medicine, University of California San Diego, 9500 Gilman Drive #0507, La Jolla, San Diego, CA 92093-0507 USA

**Keywords:** Abortion, Menstrual regulation, Family planning

## Abstract

**Background:**

In Bangladesh, abortion is illegal except to save a woman’s life, though menstrual regulation (MR) is permitted. MR involves the use of manual uterine aspiration or Misoprostol (with or without Mifepristone) to induce menstruation up to 10–12 weeks from the last menstrual period. Despite the availability of safe and legal MR services, abortions still occur in informal setttings and are associated with high complication rates, causing women to then seek post abortion care (PAC). The objective of this study is to contextualize MR in Bangladesh and understand systemic barriers to seeking care in formal settings and faciltators to seeking care in informal settings via the perspective of MR providers in an effort to inform interventions to improve MR safety.

**Methods:**

Qualitative individual semi-structured interviews were conducted with 25 trained MR providers (doctors and nurses) from urban tertiary care facilities in six different cities in Bangladesh from April to July, 2018. Interviews explored providers’ knowledge of MR and abortion in Bangladesh, knowledge/experience with informal MR providers, knowledge/experience with patients attempting self-managed abortion, personal attitudes and moral perspectives of MR/abortion in general, and barriers to formal MR. Team based coding and a directed content analysis approach was performed by three researchers.

**Results:**

There were three predominant yet overlapping themes: (i) logistics of obtaining MR/PAC/abortion, (ii) provider attitudes, and (iii) overcoming barriers to safe MR. With regards to logistics, lack of consensus among providers revealed challenges with defining MR/abortion gestational age cutoffs. Increasing PAC services may be due to patients purchasing Mifepristone/Misoprostol from pharmacists who do not provide adequate instruction about use, but are logistically easier to access. Patients may be directed to untrained providers by brokers, who intercept patients entering the hospitals/clinics and receive a commission from informal clinics for bringing patients. Provider attitudes and biases about MR can impact who receives care, creating barriers to formal MR for certain patients. Attitudes to MR in informal settings was overwhelmingly negative, which may contribute to delays in care-seeking and complications which endanger patients. Perceived barriers to accessing formal MR include distance, family influence, brokers, and lack of knowledge.

**Conclusions:**

Lack of standardization among providers of MR gestational age cutoffs may affect patient care and MR access, causing some patients to be inappropriately turned away. Providers in urban tertiary care facilities in Bangladesh see primarily the complicated MR/PAC cases, which may impact their negative attitude, and the safety of out-of-clinic/self-managed abortion is unknown. MR safety may be improved by eliminating brokers. A harm reduction approach to improve counseling about MR/abortion care in pharmacies may improve safety and access. Policy makers should consider increasing training of frontline health workers, such as Family Welfare Visitors to provide evidence-based information about Mifepristone/Misoprostol.

## Plain English summary

Abortion is illegal in Bangladesh, except to save a woman’s life. Menstrual regulation is allowed. Menstrual regulation is when someone has a procedure or takes abortion medicines when they have not had a period for 10–12 weeks. There are clinics in Bangladesh that do menstrual regulation safely, but many people try menstrual regulation outside of clinics, which sometimes causes health problems. The goal of this study was to better understand the context of menstrual regulation in Bangladesh, the barriers women face when seeking care in clinics, reasons women seek abortion care outside of menstrual regulation clinics, and to explore ways abortion care and post-abortion care can safely can be improved by asking those that provide menstrual regulation their perspective. Doctors and nurses who do menstrual regulation in large city clinics and hospitals answered questions about menstrual regulation done outside of clinics and the patients they see. They were asked how long after a missed period menstrual regulation can be done, what they know about menstrual regulation outside of the formal system, and how they feel about menstrual regulation in general and when it is done outside of clinics. They were asked about their experiences caring for women who have sought care outside of clinics, if they see barriers that keep women from accessing clinics, or if there are reasons why women choose care outside of clinics. When asked how long after a missed period the law in Bangladesh says menstrual regulation can be done, the doctors and nurses did not give the same answer, meaning some might not know the right answer. The doctors and nurses also said that since abortion medicines can be purchased from pharmacies, women are taking the medicines without being told the right way to take them and then coming to clinic with health problems. Also, women trying to get clinic menstrual regulation are sometimes taken to other places by brokers, who get money for bringing patients to these other places. Some providers do not want to provide menstrual regulation to certain patients, which can prevent some people from getting care in a clinic. Most providers also thought that menstrual regulation should not be done outside of clinics because it causes more health problems. Things that keep women from coming to clinic are being too far away from clinics, families stopping them, and not knowing about the clinics. Doctors and nurses should know the rules about menstrual regulation in Bangladesh because it might be causing some women to not receive menstrual regulation. Also, the brokers should be stopped. The doctors and nurses in this study often only see the patients that have problems, and do not see women that try menstrual regulation outside of clinics with no problems, which might cause them to think that menstrual regulation outside of clinics is bad. The best way to help women have fewer health problems might be to try to make menstrual regulation safer when it is done outside of clinics by teaching pharmacists and health workers closer to women’s homes how to provide abortion medicines and counseling about their use more safely.

## Background

Unsafe abortion is one of the leading causes of maternal mortality worldwide, and maternal deaths disproportionately occur where abortion is restricted or illegal [1]. In Bangladesh, abortion is illegal except to save a woman’s life. However, menstrual regulation (MR) has been available in the country since 1979 [2]. MR involves the use of either manual vacuum aspiration (MVA) or medications (Misoprostol with or without Mifepristone) without definitive diagnosis of pregnancy to induce menstruation and can be performed up to 12 weeks from the last menstrual period for doctors and 10 weeks for nurses [2]. Menstrual regulation is approved by the government, and safe MR services are offered within government and private health facilities [3].

Despite this formal system that allows for abortion care in a clinic, many women continue to obtain abortions outside of this system. Some of these women eventually present to clinics for post-abortion care (PAC). In 2010, approximately 231,400 women were treated for complications of out-of-clinic abortion in Bangladesh, and this is estimated to be only 40% of the total complications resulting from out-of-clinic abortion [4, 5]. In a study utilizing surveillance data from more than 100,000 pregnancies in Bangladesh, MR was found to have a lower risk of maternal mortality than live birth, while out-of-clinic abortion had a higher maternal mortality rate than live birth [6]. In this study, methods of abortion outside the health system consisted of using medications or receiving a procedure from an informal provider. It was unknown which medications were used, or if women were purchasing these medications over the counter from pharmacies to self-induce abortion [6].

A 2012 survey of healthcare facilities and providers identified that younger women with less education are more likely to have an out-of-clinic abortion in Bangladesh [5]. Systemic barriers to accessing formal MR services include provider shortages in facilities expected to provide MR, lack of equipment, or provider religious or cultural opposition [5]. Additionally, about 26% of women are turned away from MR facilities, most often due to being beyond the legal gestational age limit [3].

The government health system and various NGOs in the country have well-regulated MR clinics that provide safe care with few complications. However, due to the acceptability and accessibility of pharmacies and other informal facilities, women often do not use these well-regulated clinics as the first point of access for MR care [7]. Centralized health clinics are often seen as being more costly and difficult to access, while close-to-community providers are more familiar and trustworthy [7].

Although surveys and cross-sectional studies of MR providers have been performed, qualitative studies among trained MR providers are lacking. Qualitative assessments have the potential to lend more in-depth insight into provider experiences with women who have sought out-of-clinic abortions and their perspectives on barriers to safe abortion care. Interviewing providers can also reveal systemic barriers that might not be readily apparent to patients and other stakeholders. The aim of this study is to contextualize MR in Bangladesh and understand systemic barriers to care in formal settings and facilitators to care in informal settings via qualitative semi-structured interviews with trained MR providers in tertiary care facilities. These provider perspectives will help develop recommendations for culturally appropriate interventions that may decrease barriers and increase access to safe abortion care for women in Bangladesh.

## Methods

### Setting

MR providers, consisting of doctors and nurses, were recruited from six facilities in six out of the eight divisional (regional) capitals of Bangladesh. Facilities consisted of both medical college hospitals and the NGO-run family planning clinics attached to those medical college hospitals. The NGO-run RHSTEP (Reproductive Health Services Training and Education Program) clinics are well equipped to provide menstrual regulation, post abortion care, and contraception and their clinical services have been monitored and evaluated by Ipas. Ipas is an NGO that works to ensure safe provision of abortion care globally including through local partnerships in Bangladesh. The local study team works for Ipas Bangladesh, who have direct contact with RHSTEP, which is why the facilities were chosen. Each clinic is attached to an associated medical college hospital, which are tertiary care facilities that can handle complicated MR/PAC cases.

### Recruitment

Purposive sampling was used to focus recruitment efforts for providers in different practice settings with a diverse range of experiences with patients who attempted out-of-clinic and self-induced MR/abortion. Therefore, doctors and nurses from both RHSTEP clinics and medical college hospitals who provide MR and PAC services were recruited by study staff at regional meetings. During these meetings, all trained doctors and nurses in attendance working in the RHSTEP clinics and medical college hospitals in six of the eight divisions in Bangladesh were invited to participate in the study. The study took place from April to July of 2018. Inclusion criteria for the interviews were that the providers had to work at one of the six RHSTEP clinics or attached medical college hospitals and provide MR and PAC services. Providers were contacted to schedule a qualitative interview. Prior to the interview, informed consent was obtained by the interviewer. Qualitative interviews were conducted in a private location. Providers who completed the interviews were compensated for their pariticipation (the equivalent of $3 US according to local standards).

### Qualitative interviews

Four local Bangladeshi qualitative interviewers were trained to conduct the interviews in Bangla. In-depth interviews were individual, semi-structured, lasted approximately 1 h, and assessed providers’ knowledge of MR and abortion in Bangladesh, knowledge/experience with informal MR providers, knowledge/experience with patients attempting self-induced abortion, personal attitudes and moral perspectives of MR/abortion, and barriers to formal MR/abortion. All semi-structured interviews were audio recorded, transcribed, and translated from Bangla to English by trained research study staff in Bangladesh. Transcript files did not include personal identifiers, but were labeled with unique integer and character identifiers that were created by in-country research staff. All audio and text files remain stored in a secured multi-file location at the Ipas office in Dhaka, Bangladesh. Only English version transcripts were used for the team-based qualitative analysis that was lead by the study PI in the United States. Ethical approval of all study instruments and protocols was granted by the Bangladesh Medical Research Council (BMRC) and the University of California, San Diego Human Research Protections Program Institutional Review Board.

### Data analysis

A directed content analysis approach was used to promote a structured analytical procedure for reviewing participant transcripts [8]. A qualitative researcher (BC) began the initial cycle of open coding, making use of the interview guide to identify contextualized segments of data based on the interview questions [9]. Novel codes were created for data that could not be assigned using the question-based coding schema [10]. A team-based approach was used for the second coding cycle to resolve discrepancies in assignment or description of codes by discussion and consensus among members of the research staff (BC, ST, and SH) [11]. Interview transcripts were uploaded along with the codebook to Dedoose [12] (Version 8.0.42, 2019), a web-based qualitative analysis program. This program has flexible features to facilitate organizing and sorting the transcripts, as well excerpting quotes, and locating key words and phrases responding to the original interview guide. Using Dedoose and the final draft codebook, a third cycle of review assisted in merging some of the earlier excerpts and into nested categories because of conceptual similarity or redundancy, while other codes were dropped due to lack of utility to the specific aims of the study (BC, ST, and SH). At the conclusion of coding, we extracted all of the coded excerpts and refined their organization by framing the coding patterns, interconnections and overarching themes in the study data (BC, ST, and SH).

## Results

A total of 25 trained MR providers who worked in medical college hospitals and RHSTEP clinics were initially recruited, and all consented to participate in the semi-structured interviews. Twelve nurses and 13 doctors participated. There were 19 providers from medical college hospitals and 6 providers from RHSTEP clinics. The six divisions were relatively equally represented. Four providers from each division were interviewed, except one division which had five providers interviewed.

There were three predominant yet overlapping themes that providers used to discuss MR, PAC and abortion care in Bangladesh:

i. Logistics of MR/PAC/abortion in Bangladesh: MR providers defined MR, PAC, abortion, and gestational age cutoffs. They detailed their experiences treating patients that have sought out-of-clinic and self-managed abortion. They described the logistics of obtaining MR/PAC/abortion and who is providing them.
ii. Attitudes: MR providers detailed their own attitudes about MR in general, informal MR providers (including pharmacists), and self-management of abortion.
iii. Overcoming Barriers: MR providers outlined perceived barriers to formal MR and gave recommendations for interventions to overcome those barriers.

Interwoven among these themes were the impact of various factors on patient autonomy when deciding to have MR or abortion.

### Logistics of MR, PAC, and abortion in Bangladesh

#### Gestational age cutoffs

There was a lack of consistency among trained MR providers about appropriate or legal gestational age cutoffs for receiving MR care. This lack of consistency was present among all sites, practice settings, and provider roles. There was no standardization among the providers as to how far along MR or abortion could be performed or if abortion can actually be performed in Bangladesh:

Actually there is no abortion in Bangladesh, the term is MR…Some patients tell straightway, “I have come here for abortion.” Then we ask them how many months her period is stopped? Suppose she replies, 2 or 2½ months, then we make her correct that she has come for regularizing her menstruation (Nurse 2, RHSTEP).

In contrast, another doctor stated, “In Bangladesh we are doing abortion; we termed it as abortion if it is occurred within 28 weeks…those who are under 12 weeks, we do MR services to them. We can do MR until 9–12 weeks. But those who are non-doctors or trainees, they can do MR within 9 weeks” (Doctor 11, medical college hospital). Several providers acknowledged that MR can be performed up to 10–12 weeks depending on the type of provider, but there were many deviations from this standard. According to one nurse, “Abortion can be done even at 35 weeks…MR can take place only within 14 weeks” (Nurse 8, medical college hospital). Another nurse stated, “Abortion can happen in 8 weeks or 12 weeks and even in 14 weeks but you have to do MR within 8 to 10 weeks. If it is 12 weeks, then it is risky” (Nurse 10, medical college hospital). One doctor said, “[MR can be done before] seven menstrual weeks” (Doctor 6, medical college hospital). One RHSTEP doctor stated, “MR is generally 10 weeks for doctors and 8 weeks for nurses” (Doctor 8, RHSTEP). If providers are unaware of the legal gestational age cutoffs for MR, it may contribute to patients begin turned away from facilities, even if they are within the legal gestational age limit.

#### Logistics and facilitators to care from informal out-of-clinic providers

According to providers, patients sometimes are referred to informal out-of-clinic providers by brokers and via word-of-mouth from family and community members. Brokers intercept patients entering the hospitals/clinics for MR or PAC and receive a commission from informal clinics for bringing patients. Although some traditional healers and homeopathic providers perform out-of-clinic abortions, a lot of the informal providers have previously worked in the hospitals and formal clinics as observing assistants but have not usually received formal training:

These service providers may have gained some experience by watching the service delivery process, for example, this aunty has become an expert by watching MR regularly here. Like her, these service providers might have worked for a center and then they decide to open a private clinic by their own (Doctor 13, medical college hospital).

Do you know that some of our Ayas [sisters/untrained lay hospital workers], they introduce themselves as Nurse to the patients and take them? But they are not nurse, they do not have any training (Nurse 6, medical college hospital).

The situations in which these informal providers practice were described as unhygienic without proper sterilizing equipment, often located in the provider’s private home. One doctor explained:

The official environment and the outside environment are not the same. We maintain a lot of things officially but it is not possible to maintain it in the home. We do autoclave regularly our instrument. We don’t use any instrument twice without autoclave. But those service providers don’t do that. They wash the instruments with water and use it again and again (Doctor 3, RHSTEP).

The methods used to induce MR/abortion can vary by the type of provider. Traditional healers tend to use herbs and tree roots, though providers report this is becoming less common:

Previously we got many patients who have been maltreated by the traditional healers. They used herbs and indigenous methods…Nowadays these types of clients has reduced…Some patients arrived [at the] hospital with septic infections and herbal roots inserted into their uterus (Nurse 2, RHSTEP).

More commonly reported was use of manual vacuum aspiration (MVA), as “MVA set is available to purchase…They perform with the instruments in this way” (Doctor 4, medical college hospital). Some informal providers perform abortion procedures “by hand-curette” (Nurse 3, medical college hospital).

Providers at RHSTEP and in the medical college hospitals come to know of these informal providers because they treat patients who have complications, which creates unfavorable opinions of the informal providers. They describe that some patients come after seeing informal providers with “…septicemia, septic abortion, incomplete abortion…Today, we had a patient [in which] part of the digestion system was cut off during MR…We had to perform surgery on her. These types of issues are horrific” (Doctor 2, medical college hospital). Other providers acknowledge that some out-of-clinic MR could be performed without complication, as “those who get performed without complications, they do not come to us. They go back from there. Those who face complications they come to us” (Doctor 4, medical college hospital).

#### Logistics and facilitators to self-managed abortion

With regards to self-management of abortion, providers indicate that although traditional methods provided by herbalists and traditional healers are still being used, using Misoprostol (with or without Mifepristone) is being more commonly reported, which is often purchased over-the-counter in pharmacies. Patients obtain methods “through others, friends and relatives and also from the brokers….The family members instruct them. Husband goes to the medicine shop and told the drug-seller that his wife missed her period…Then the drug seller tells that if she takes 4–5 Misoprostol, it will solve the problem” (Nurse 1, medical college hospital). Traditional methods include herbs and “medicine made from grass root. [One patient] put this medicine in cervix…she poked [back and forth] with it in the uterus. Many people think that if they put pressure in abdomen fetus can expel” (Doctor 5, RHSTEP). Often, providers described patients trying several different methods to self-manage abortion before finally presenting to a formal clinic, though one commonly reported method was medications purchased over the counter from pharamacies without a prescription. The providers also noticed a change in the number and type of services they provide due to these practices:

Some take medicine; some go to the [untrained providers]. In [many] cases those who come to us have tried all these methods. First they take homeopathic, then go to the kabiraj (herbalist) and go to the [untrained providers]. When it becomes incomplete, then they come to us (Doctor 7, medical college hospital).

MR services [are] decreased. You can get misoprostol pills at pharmacy and everyone is using it…They do not need anything to buy it from pharmacy. Pharmacy gives the medicine to everyone like to the patients or to the husbands (Doctor 5, RHSTEP).

Providers are aware of self-management practices because they treat patients with complications which creates unfavorable attitudes about self-management. Complications from self-management include “incomplete [abortion and] excessive bleeding…Another problem is that even though they do it, the pregnancy continues. They say that they had taken medicine but it continued [to] 16–18 weeks” (Doctor 9, medical college hospital). Another provider encountered a patient “with ectopic pregnancy! She came here after taking medicine advised by her neighbor. But, she needed an open surgery… Some of them who are taking medicine from the pharmacists are having incomplete abortion” (Nurse 2, RHSTEP). Many complications from pharmacies are thought to be due to inadequate screening and instruction:

The MR through medicine also needs doctor’s instructions. We have to tell them about the dose, its use, through which route it would pass, the complications that may arise, full warning signs. They will not get such instructions from the pharmacy (Doctor 10, RHSTEP).

Other providers acknowledge that, again, they are only seeing the complications from self-managed abortion, and other cases may be happening without complication. One provider knows “about two to three persons who have done it by themselves without any problem. It was complete” (Doctor 10, RHSTEP).

### Spectrum of provider attitudes

#### MR in general

There were a variety of attitudes among trained MR providers with regards to the ethics and practice of MR, and ethics was often tied to religion. Providers working for RHSTEP tended to have a more positive view of MR, explaining the value of MR services and the benefit to patients. They also tended to respect patient autonomy and did not change practice based on the patient’s social or family situation. One RHSTEP provider explained, “[MR] is useful for [many] girls. Otherwise many girls had to commit suicide or suffer a lot” (Nurse 2, RHSTEP). Some providers in medical college hospitals also had positive views and respected patient autonomy in decision making:

From religious point of view I had some [objections]. But when I got the training of PAC and MR from IPAS, my view had changed. That training was positive for me. Now I always provide this service to the patients (Doctor 1, medical college hospital).

[I tell patients when deciding on MR] Even husband’s confirmation is not needed; your right is the main. You have to do which is right for you. You have to think about your health, your in laws will not think about it (Nurse 11, medical college hospital).

However, when providers have negative attitudes about MR or impose their own moral judgement on the patient’s decision, patient autonomy can be affected, creating barriers to MR. Some providers had religious hesitations to providing MR, and others would only perform MR in certain situations:

Usually we do not try to give MR and PAC services to the patients. First of all, we see how many children they have, how much her demand is… If we see her child age is one year or 8/9 months old then we think about MR and PAC services for her because these are good for her as her child is very much young for taking another child (Nurse 7, medical college hospital).

I think that one should not go for a MR. This would be advantageous for all. We are Muslims. It is not the question of a Muslim only, in fact, every religion forbids killing a life (Nurse 8, medical college hospital).

Other providers will only perform MR “if there is husband, mother-in-law, or mother with them” (Doctor 7, medical college hospital). If providers refuse to perform MR, this creates barriers, causing women to seek care from other informal sources. According to one nurse:

She has to do an abortion anyhow. It can be by doctor or nurse. But sometimes doctors or nurses do not give her any counseling and blame her to be pregnant because she is unmarried and ignore her. That time she goes to inexpert midwife or quack (Nurse 10, medical college hospital).

#### Patients seeking MR

Providers sometimes judged patients for their decisions, especially if they sought an out-of-clinic abortion. One provider described punitive behavior toward patients presenting with complications from out-of-clinic abortion:

We then tell them, “Why have you delayed? Why have you done this?”…They need blood but will not come to hospital. “Now you have come when you are about to die.” We rebuke them like this when they come with problem (Nurse 6, medical college hospital).

Other providers judged patients who seek MR in general as having lower education, affecting how providers view their decision making capacity. This was common throughout all provider roles and practice settings. One doctor explained, “Educated people are not much to come for MR, they are conscious. MR services take place around mostly lower class” (Doctor 3, RHSTEP). Another provider thought lower class women had different motivations than other women, stating, “Women do not want to destroy [their] baby – those who want it – it is done by the slum dwelling women…They want to do it. But directly no woman wants to destroy her baby” (Nurse 1, medical college hospital).

### Overcoming barriers to formal MR

#### Identifying barriers

Providers identified several barriers to obtaining formal MR care, including logistical, cultural, provider/health facility related barriers, and brokers.

#### Logistical barriers

Logistical barriers include cost, lack of knowledge, and family obligations. When seeking MR, “the hospitals seem distant for [the clients], and they think about the expense that will occur because of the distance [so] they get a sister [untrained lay health worker] beside them who does the MR” (Doctor 1, medical college hospital). Furthermore, clients “may not be aware of service. As you know sister, in many villages these messages have not reached yet” (Nurse 6, medical college hospital). With regards to family, husbands and mothers-in-law often dictate the care that a woman receives, and family obligations require the woman to stay close to the home. Opinions of the family can affect patient autonomy. One nurse spoke of the consequences of disobeying the mother-in-law:

Some in-laws say like “Do not go there. It will work out if you take herbs, if you drink holy water…” Then they will go to the religious leader [and] traditional healers. When they come they say that their mothers-in-law were creating obstructions for them…[but] if they do not obey their mothers-in-law…maybe they will be divorced, they will be thrown out of their house, their in-laws will arrange another marriage (Nurse 6, medical college hospital).

Another doctor discussed that women’s family obligations make it difficult to be away for longer periods of time:

Many feel that coming to the hospital means staying here for 2-3 days…that’s why they seek service in her locality from a quack doctor or a traditional healer or known health assistant working in a nearby health complex…In addition, if she gets anything done in the locality, she can also look after her family (Doctor 10, RHSTEP).

#### Cultural barriers

Additionally, religion can create a cultural barrier to MR in general, though there were varying opinions on if this affects where women present for care. Some providers unequivocally stated that “There are religious obstacles…especially the Muslims face religious obstacle” (Nurse 11, medical college hospital). However, one doctor in Dhaka explained:

There are no religious obstacles to come into the facilities. Even the wife of a Huzur [Muslim religious leader] is also coming to us for MR as well as the wives of a Moulana or Imam…If they think about it, they would not attempt MR (either in facilities or anywhere)…The problem is that they are scared that everyone will know that they come to the centre. They don’t want to let anyone know about this incident (Doctor 1, medical college hospital).

Other providers indicated that religious opinions of MR may not be homogeneous, varying with different levels of knowledge and conservativism:

There are some types of people but they are not real huzur. Who are real huzur, they understand these issues, and they are conscious persons…However, who are fanatical they don’t’ allow the wife to go to facilities as there are large gatherings at facilities (Nurse 7, medical college hospital).

Religion can be a barrier for MR in general, and in certain circumstances depending on the level of religious conservativism, may affect if a woman can receive care in public settings.

#### Health facility/provider barriers

Furthermore, providers discussed that there are aspects of the health centers and the providers themselves that create barriers to in-clinic MR. Time constraints and provider attitudes cause patients to seek care elsewhere, especially in government health facilities:

One physicians supposed to see 200 – 300 patients within 6 hours. The same physician…in a private clinic, he has to see 5 – 6. He gives 20 minutes to 30 minutes to each patients in a private clinic…In [other] centers many patients come for service but the providers don’t provide services at all (Nurse 2, RHSTEP).

Providers also recognize that their own behavior can deter patients from coming to health centers. One provider explained, “We doctors don’t want to do counselling as sometimes women don’t understand after explanation. Because of this the patients don’t understand what has happened to them… we also cannot explain properly due to lack of time. There is a gap here” (Doctor 8, RHSTEP).

#### Brokers

Overwhelmingly, providers of all roles from all different practice settings and sites indicated that brokers create immense barriers for patients in seeking in-clinic MR. Brokers intercept patients seeking MR from RHSTEP or the government health facility and take them to other clinics, which are often informal, where they receive a commission:

They maintain a strong syndicate from the main gate of the hospital…They convince patient to go the private clinic to receive better quality medical service compare to government hospital. The service receivers go to the private clinic and take treatment facilities from [a] quack (Doctor 12, RHSTEP).

Brokers not only stand outside the hospital gate, but can be members of the hospital staff as well, charging patients for referrals. One nurse said that “the maids, ward boys, they are brokers…they are taking one thousand taka from [patients]” (Nurse 12, medical college hospital). Since they may be members of hospital staff and health centers can be large and difficult to navigate, patients often trust the brokers to take them to the correct location, though they end up in informal clinics. Even if patients become suspicious of brokers, once they are in another informal clinic, they sometimes have to pay just to leave:

[One woman] was searching for RHSTEP clinic. A broker took her to another clinic. Seeing the environment…the patient assumed that something is wrong. She told that broker, “…I don’t want to stay here, I want to go back.” The broker then said, “…You have to pay before you leave otherwise we won’t let you leave” (Nurse 2, RHSTEP).

In this case, the broker created a barrier to in-clinic MR and affected the woman’s autonomy since she was not taken to her choice of provier.

#### How to improve MR care

When discussing how MR care can be improved in Bangladesh, providers suggested further education/outreach, training, increasing services in more peripheral settings, and punitive action against brokers. Some providers recommended education of villagers about MR services via meetings and leaflets. Although some advocated for punishing untrained providers, others advocated for further training, stating “It would be better if those [untrained providers] could actually be identified. And it would be best if they could be provided with training…As you know, sisters can be trained” (Doctor 4, medical college hospital). One nurse also suggested training providers in appropriate behavior:

When you do adequate counselling then the patient will go and tell that [this provider] is so good, she spoke to me nicely. Behaviour is also very good…and she would speak well of us. She would go back and propagate (Nurse 5, medical college hospital).

Although there are some providers doing MR in more rural settings, many providers thought that care outside of the tertiary care centers could be improved and expanded to include community based trained frontline health workers called family welfare visitors (FWV):

There are also some trained nurses who can do this kind of service. They are also working in periphery. We can train the FWV who are working in the sub centres. At least they have some knowledge and they will act accordingly (Doctor 1, medical college hospital).

Finally, many providers wanted punitive action against the brokers:

If any journalist would help [with the brokers], investigate this issue placing self as a patient, then these criminals would have been exposed…These people are harassing these MR patients, this should be stopped (Nurse 2, RHSTEP).

## Discussion

Many women in Bangladesh seek MR services outside of formal clinical settings, whether it is from other providers or self-management of abortion. Although women still visit informal providers and traditional healers, who frequently use herbs, foreign objects in the cervix/uterus, or MVA or sharp curette, MR providers thought that the practice of taking medications provided by pharmacies has become more common. Complications encountered by providers in tertiary care settings include bleeding, infection, retained products of conception, failure to end pregnancy, uterine perforation, and sometimes death, causing most providers to have negative attitudes about informal providers and pharmacists offering medication abortion. Other barriers to clinic MR cited by providers included cost, distance, lack of knowledge of services, family obstruction, religion, and provider behavior. The overwhelming majority of providers especially emphasized that brokers are a barrier to clinic MR care and create unsafe care for patients. Although the MR providers in this study received formal training, there was a lack of consistency about gestational age cutoffs for performing MR legally.

 The perspectives of trained MR providers in this study are consistent with several other studies on MR/abortion in Bangladesh. Similar to other studies, the providers in our study described that complications of out-of-clinic abortion are still higher than in-clinic MR, though the use of less safe methods, such as foreign objects in the cervix/uterus and sharp curette are decreasing over time [3, 4, 6, 13]. The description of pharmacists providing inadequate instruction when prescribing Mifepristone and Misoprostol is also consistent with prior studies [14]. Footman et al. followed MR clients seeking medications from pharmacies and found that 40.4% of clients received no instruction, and only 8.7% received written information or pictures [15]. Many pharmacists lack formal medical training, which leades to decreased quality in service provision, including the safe provision of medication abortion in Bangladesh [16]. For example, during mystery client visits to pharmacies, Huda et al. found that only 54% of pharmacists provided the recommended dosage of Mifepristone and Misoprostol, and only 11% provided any counseling on possible complications [17].

Other studies have also cited cost, distance, lack of knowledge of services, family obligations, long wait times at government hospitals, and perceived lack of quality of care at hospitals as creating barriers or delays in obtaining MR in formal clinics [3, 7]. They have also described similar facilitating factors of pharmacies and close-to-community providers, such as less cost and less of a need for transport, when seeking MR services [7]. When discussing how MR services can be improved, MR clients have agreed with the viewpoints of the providers in this study that better instruction and treatment of patients needs to be provided, and services need to be more readily available in peripheral settings [18]. The Ministry of Health in Bangladesh has prioritized increasing the number and responsibilities of frontline health workers, such as FWVs, to supplement family planning care in more rural areas [19]. Expanding the scope of FWVs to include MR with Mifepristone and/or Misoprostol may improve out-of-clinic abortion safety.

One finding of this study is that provider attitudes may affect access to MR care and undermine patient autonomy. The definition of quality contraceptive counseling articulated by Judith Bruce in 1990 not only includes respecting patient dignity and autonomy, but also increasing access to family planning, and recent revisions have promoted expanding this model to all aspects of reproductive health [20]. Expanding these guidelines to MR services in Bangladesh may also increase access to safe MR services for patients. Additionally, as self-management via medications from pharmacies is becoming more common, training providers to support patients by providing non-judgemental patient-centered post-abortion care may further facilitate patients seeking care in the future or referring others in their communities. Studies have previously mentioned that although certain providers are trained in MR, they may not be practicing MR due to their personal or religious beliefs [21]. The providers in this study alluded to certain scenarios in which they would not perform MR or would try to encourage a patient to not seek MR, such as when a husband/mother-in-law were not present or if a married patient did not yet have children. Furthermore, Providers commonly described patients receiving MR as being of a lower class or less educated, which may also affect how they view clients’ ability to make autonomous decisions about their pregnancy intention.

Although previous studies have described that patients are turned away from MR facilities, the main reason described in Hossain et al. was not provider attitudes but being beyond the legal gestational age limit [3]. In that study, most women thought they were 3–4 months pregnant at the time of seeking MR, though the exact gestational ages are unknown, and the most common gestational age cutoff for MR given was 12 weeks [3]. A surprising finding of this study was the lack of standardization among trained MR providers of gestational age cutoffs for performing MR legally. Historically, doctors were able to perform MR until 10 weeks, and mid-level providers were able to perform MR until 8 weeks. However, in 2014, a law was passed allowing doctors to perform MR until 12 weeks, and mid-level providers until 10 weeks [2, 22]. The varying gestational age cutoffs could not be described by different provider cadres, as the reported range in which MR could be performed varied between 7 and 14 weeks, though may indicate that many providers might not be educated about the law update. This discrepancy persisted among all provider roles, all practice settings, even in certain RHSTEP clinics, whose providers have been formally trained by Ipas. However, follow up training may be needed. In the medical college hospitals, providers may not have received formal Ipas training and may have been trained through the government medical education system, explaining some of the discrepancy. In Hossain et al., most women were not aware of the legal gestational age cutoff for MR [3]. If there is inconsistent provider knowledge of gestational age cutoffs, it may make it difficult to provide education about legal MR cutoffs, and patients may be turned away even if they are within the legal MR window. This study highlights that further training of MR providers is needed.

Furthermore, one barrier to formal MR (and safe MR in general) that was repeatedly highlighted by providers in this study is the presence of brokers at medical college hospitals and RHSTEP clinics. Brokers have been briefly mentioned in other studies, but the fact that most providers from all clinical settings and all provider roles described their detriment to patient care was a novel finding in this study. One qualitative study previously mentioned brokers in focus group discussions with community members about seeking general sexual reproductive healthcare [7]. Some focus group participants seemed to think that a broker was necessary in order to obtain a doctor’s appointment and that it was not possible to access the doctor or obtain a hospital admission directly [7]. The sentiment among some community members was that doctors in the government health center and brokers are working together. However, the MR providers in this study clearly see brokers as a barrier to accessing formal MR services and believe that the brokers re-direct patients to unsafe care by taking them to informal clinics where the providers may not be trained and the facilities may not be clean or the equiptment sterile. There was no indication of collaboration between these trained MR providers and brokers, and many providers thought that the brokers should be punished.

### Strengths and limitations

The strengths of this qualitative study are that it outlines out-of-clinic and self-managed abortion from the perspective of experienced MR providers in tertiary care settings who routinely treat both MR/PAC patients and complications from out-of-clinic abortion. The large number of interviews performed (25) across a variety of divisions in Bangladesh ensured that the sample size was representative of doctors and nurses in tertiary care facilities. The interviews were individual and confidential to allow for providers to give their honest and intimate perspectives about a sensitive topic.

A limitation of this qualitative study is that it is not generalizable to all MR providers in Bangladesh. The providers interviewed work only in tertiary care settings within urban divisional capitals. Because of this, they often witness the most complicated MR cases, which may cause them to have a more negative opinion of informal providers, pharmacists, and self-management of abortion. Additionally, providers in this study have limited experience with informal providers and self-management of abortion when it happens safely without complications since those patients are not seen for their care. Another limitation of the current study is that, with the exception of the providers who have performed site visits to investigate informal clinics, most providers have not directly witnessed the conditions and quality of care of informal providers/pharmacists and only know through patient report and word-of-mouth. With regards to qualitative methodology, one limitation is that the semi-structured interviews were not performed by the qualitative researchers responsible for the data coding and analysis, which would have been ideal. However, the study team felt like the interviews needed to be performed by individuals of a similar cultural background to increase cultural concordance, and interviews were performed by local professionals trained by the in-country team. To account for this, team coding was performed to promote discussion and consensus, addressing any discrepancies in code assignment or description. The qualitative researchers maintained contact with the in-country interviewers, providing feedback on interview and transcription techniques and asking clarifying questions of translations. Finally, there is little demographic information known about the providers that were interviewed, such as age, religion, and years in practice. Given the senstitive nature of the topic and potential stigma for providing care, little demographic information was collected to maintain confidentiality. However, provider responses may be impacted by confounding characteristics not recognized in this study, especially religious beliefs and the varying levels of conservativism within religion.

## Conclusion

MR care in Bangladesh can be improved by increasing community education about MR, broadening the provider base for formal MR provision to include pharmacists and possibly FWVs, decentralizing services/expanding types of facilities where MR can be provided (such as pharmacies and in the community), and taking action to stop the brokers. Provider training and increasing access in decentralized facilities could be achieved via increasing training of frontline health workers, such as FWVs to provide evidence-based information about Mifepristone/Misoprostol. The safety of self-managed abortion may be improved by a harm reduction approach to improve MR/abortion care provided by pharmacies. Additionally, policy makers should develop penalties for brokers who lead patients to seek unsafe care. Further studies are needed to provide a more robust picture of out-of-clinic and self-managed abortion in Bangladesh. Qualitative studies among providers in more rural or peripheral clinical settings would be beneficial to assess the quality of services, barriers and facilitators to improving MR services in these peripheral settings, and their experiences with complications. Qualitative studies with informal providers about MR/abortion care specifically would also be helpful in obtaining a more direct depiction of their services, their experiences with complications, and their willingness and capacity to receive further training. Finally, interviews with MR clients seeking care out-of-clinic would provide further insight into their abortion experiences and barriers/facilitators of out-of-clinic and self managed abortion, especially among clients who have not experienced complications. Interviews with clients who have experienced complications of out-of-clinic abortion would provide insight on how to improve its safety.

## Data Availability

Data sharing is not applicable to this article as no datasets were generated or analysed during the current study.

## Abbreviations

MR: Menstrual regulation
PAC: Post-abortion care
MVA: Manual vaccum aspiration
D&C: Dilation and curettage
RHSTEP: Reproductive Health Services Training and Education Program
NGO: Non-governmental organization
SSIs: Semi-structured interviews
FWVs: Family Welfare Visitors
